# Supplementary material for: Pathways of association between maternal haemoglobin and stillbirth: path-analysis of maternity data from two hospitals in England
Source: BMJ Open. 2018 Apr 7;8(4):e020149. doi: 10.1136/bmjopen-2017-020149 (PMC5892753; doi:10.1136/bmjopen-2017-020149)
Supplement: Author's manuscript [file bmjopen-2017-020149.draft_revisions.pdf]

BMJ Open is committed to open peer review. As part of this commitment we make the peer review history of every article we publish publicly available.

When an article is published we post the peer reviewers' comments and the authors' responses online. We also post the versions of the paper that were used during peer review. These are the versions that the peer review comments apply to.

The versions of the paper that follow are the versions that were submitted during the peer review process. They are not the versions of record or the final published versions. They should not be cited or distributed as the published version of this manuscript.

BMJ Open is an open access journal and the full, final, typeset and author-corrected version of record of the manuscript is available on our site with no access controls, subscription charges or pay-per-view fees (http://bmjopen.bmj.com).

If you have any questions on BMJ Open's open peer review process please email info.bmjopen@bmj.com

BMJ Open

Pathways of association between maternal haemoglobin and stillbirth: path-analysis of maternity data from two hospitals in England

Journal:	*BMJ Open*
Manuscript ID	bmjopen-2017-020149
Article Type:	Research
Date Submitted by the Author:	17-Oct-2017
Complete List of Authors:	Nair, Manisha; University of Oxford, NPEU, Nuffield Department of Population Health<br>Knight, Marian; National Perinatal Epidemiology Unit<br>Robinson , Susan ; Guy's and St Thomas' NHS Foundation Trust<br>Nelson-Piercy, Catherine; Guy’s & St Thomas’ Foundation Trust<br>Stanworth, Simon; Oxford Radcliffe Hospitals Trust, Department of Haematology/Transfusion Medicine<br>Churchill, David; The Royal Wolverhampton Hospital NHS Trust, New Cross Hospital
**Primary Subject Heading**:	Epidemiology
Secondary Subject Heading:	Public health, Obstetrics and gynaecology
Keywords:	Haemoglobin concentration, pregnancy, stillbirth, path-analysis

SCHOLARONE™
Manuscripts

Title page

Pathways of association between maternal haemoglobin and stillbirth: path-analysis of maternity data from two hospitals in England

AUTHORS

1. Manisha Nair
National Perinatal Epidemiology Unit (NPEU), Nuffield Department of Population Health, University of Oxford, Richard Doll Building, Old Road Campus, Headington, Oxford, OX3 7LF, United Kingdom; Email: manisha.nair@npeu.ox.ac.uk

2. Marian Knight
NPEU, Nuffield Department of Population Health, University of Oxford, Richard Doll Building, Old Road Campus, Headington, Oxford, OX3 7LF, United Kingdom, Email: marian.knight@npeu.ox.ac.uk

3. Susan Robinson
Guy's and St Thomas' NHS Foundation Trust
Guy's Hospital, Fourth Floor Southwark Wing, Great Maze Pond, London, SE1 9RT; Email: Susan.Robinson@gstt.nhs.uk

4. Cathy Nelson-Piercy
Guy's and St Thomas' NHS Foundation Trust
10th floor North Wing, St Thomas' Hospital, Westminster Bridge Road, London SE1 7EH; Email: Catherine.Nelson-Piercy@gstt.nhs.uk

5. Simon J Stanworth
Oxford University Hospitals NHS Foundation Trust/ NHS Blood and Transplant
John Radcliffe Hospital, Headley Way, Oxford OX3 9DU; Email: simon.stanworth@nhsbt.nhs.uk

6. David Churchill
The Royal Wolverhampton Hospital NHS Trust, New Cross Hospital, Wolverhampton, WV10 0QP; Email: david.churchill1@nhs.net

Corresponding author

Manisha Nair
National Perinatal Epidemiology Unit (NPEU), Nuffield Department of Population Health, University of Oxford, Richard Doll Building, Old Road Campus, Headington, Oxford, OX3

For peer review only - http://bmjopen.bmj.com/site/about/guidelines.xhtml

7LF, United Kingdom

Phone: 01865-617820

Email: manisha.nair@npeu.ox.ac.uk

ABSTRACT

Objective: To investigate the mechanisms that link maternal haemoglobin concentration with stillbirth.

Design: A retrospective cohort analysis using anonymised maternity data from two hospitals in England.

Setting: The Royal Wolverhampton NHS Trust and Guy's and St Thomas' NHS Foundation Trust

Study population: 12,636 women with singleton pregnancies ≥24 weeks of gestation giving birth in the two hospitals during 2013-15.

Method: A conceptual framework of hypothesised pathways through birthweight-for-gestational age and maternal infection including potential confounders and other risk factors was developed and examined using path-analysis. Path-analysis was performed by fitting a set of regression equations using weighted least squares adjusted for mean and variance. Goodness-of-fit indices were estimated.

Main outcome measures: Coefficient of association (β) for relationship between each parameter, and direct, indirect and total effects via the postulated pathways.

Results: The path-model showed a significant adjusted indirect negative effect of maternal haemoglobin on stillbirth mediated via birthweight-for-gestational age (Standardised estimate (SE) = -0.01; 95% CI = -0.01 to -0.001; p=0.028). The effect through maternal infection was not significant at p<0.05 (SE= 0.001; 95% CI = -0.004 to 0.01; p=0.610). There was a residual direct negative effect of maternal haemoglobin on stillbirth (SE = -0.12; 95%CI -0.23 to -0.02; p=0.020) after accounting for the two pathways. Total indirect SE = -0.004; 95% CI -0.01 to 0.003; p=0.267; total direct and indirect SE = -0.13; 95% CI -0.23 to -0.02; p=0.016. The goodness-of-fit indices showed a good fit between the model and the data.

Conclusion: While some of the influence on risk of stillbirth acts through low birthweight-for-gestational age, the majority does not. Several new mechanisms have been suggested for how haemoglobin may be exerting its influence on the risk of stillbirth possibly involving genetic, epigenetic and/or alternative obstetric and nutritional pathologies, but much more research is needed.

ARTICLE SUMMARY

Strengths and limitations of the study

- While a number of studies have demonstrated low maternal haemoglobin to be a risk factor for stillbirth, this study advances the field by delineating the pathways through which maternal haemoglobin could affect stillbirth.

For peer review only - http://bmjopen.bmj.com/site/about/guidelines.xhtml

- Using path-analysis we quantified two theoretical pathways through which maternal haemoglobin could affect stillbirth: (i) mediated via birthweight-for-gestational age and (ii) via maternal infection.
- It is important to acknowledge that path-models are not causal models and therefore the findings of this study are hypothesis-generating rather than demonstrating causal pathways.
- Inability to adjust for socioeconomic status in the main model was a limitation, but sensitivity analysis using the Wolverhampton data did not materially change the results.
- Information about the causes of stillbirth in the study population was not available and it is possible that the mechanisms through which haemoglobin affects stillbirth vary by cause.

Key words: Haemoglobin concentration, pregnancy, stillbirth, path-analysis

Word count: 2980

For peer review only

INTRODUCTION

Stillbirth is a global problem, and while the rate has been gradually falling it is difficult to discern the impact of different initiatives. In high income countries, the rates have only marginally improved. Many of the risk factors associated with stillbirth are known and include maternal obesity, advanced maternal age, smoking, small for gestational age fetuses, placental abruptions, placental pathology, pre-existing diabetes and hypertension[1]. Many initiatives have been and are being deployed to try to modify these risk factors and to reduce the rate of stillbirth further, but so far, the improvements have been small, for a combination of reasons. Risk factors such as smoking and obesity are hard to modify in a short period of time and ideally, to have a significant impact, need to be influenced in the peri-conception period. Likewise, in the instance of pre-existing diabetes, it has been shown that pre-conception blood glucose control is as important as good blood glucose control during pregnancy[2 3]. Other risk factors are pathologically non-specific. Small for gestational age, as applied to the fetus, selects a population who are considered at risk. It does not detect the 'sick' fetus. Other tests such as assessments of fetal wellbeing need to be employed to do that, but they too have their diagnostic limitations. Risk factors such as abruption are at the end point of the natural history of disease and are a manifestation of established placental pathology. As such modifying the processes that lead to fetal death from these stand points is difficult. The main and often only intervention available to the obstetrician is to deliver the baby, which is an intervention that can cause significant morbidity and rarely mortality, for either the mother and/or fetus. As a result, calls have been made to improve the understanding of the epidemiology and the causal pathways of stillbirth so that interventions can be targeted at points where the causes of stillbirth are amenable to modification[4].

We recently studied the association between maternal anaemia and stillbirth in a cohort of 14001 pregnant women[5]. The cohort was drawn from two inner city populations in England. After adjusting for 11 known confounding variables, the risk of stillbirth decreased linearly per unit (10g/l) increase in haemoglobin concentration measured in the first trimester visit between 9 and 12 weeks; aOR = 0.70, 95% CI 0.58-0.85. Compared with women who had a haemoglobin concentration of over 110 g/L in the first trimester, the risk of stillbirth was 5-fold higher in women with moderate to severe anaemia, <100 g/L. The objective of this study was to further investigate the mechanisms that link maternal haemoglobin concentration in the first trimester of pregnancy with stillbirth.

METHOD

Study design

For peer review only - http://bmjopen.bmj.com/site/about/guidelines.xhtml

We conducted a retrospective cohort analysis using anonymised maternity data from 14,001 women with singleton pregnancies ≥24 weeks of gestation giving birth in two hospitals between 2013 and 2015 (7,175 from Royal Wolverhampton NHS Trust, 2013-14 and 6,826 from Guy's and St Thomas' NHS Foundation Trust, 2014-15). Information on maternal haemoglobin concentration at the first visit, usually between 9 to 12 weeks' gestation was extracted from the hospital laboratory databases and then paired with maternity data. The datasets were then anonymised and analysis was restricted to 12,636 singleton babies born after 24 weeks of gestation and for whom information about haemoglobin at first visit and infant outcomes was available. A theoretical conceptual framework of the hypothesised pathways through which maternal haemoglobin could be associated with stillbirth was developed using Directed Acylic Graphs[6 7] (shown in Figure 1). This was subsequently tested using a statistical modelling technique known as path-analysis.

Conceptual framework

It has been shown that causal pathways for stillbirth involve fetal growth restriction[8]. Several studies show that maternal haemoglobin concentration is inversely associated with fetal growth restriction leading to a higher risk of small for gestational age babies in pregnant women with anaemia[9 10]. Also, low haemoglobin concentration is known to be associated with increased risk of infection during pregnancy and delivery. Therefore, it was hypothesised that the observed effect of haemoglobin concentration on stillbirth could be potentially mediated via birthweight-for-gestational age (an indicator for fetal growth restriction) and/or maternal infection during the index pregnancy. Major factors identified from the literature that could confound the association between maternal haemoglobin and stillbirth were pregnancy induced hypertensive disorders, ethnicity, antepartum haemorrhage, low socioeconomic status, and medical comorbidities. In addition, although not directly associated with maternal haemoglobin, factors such as smoking, high body mass index (BMI), nulliparity, advanced maternal age (>35 years), gestational diabetes and pre-existing diabetes mellitus[8 11 12] are also important as risk factors for stillbirth and therefore were included in the conceptual framework (Figure 1).

Study variables

Socio-demographic characteristics, BMI, obstetric history, current pregnancy problems, and medical co-morbidities were used to generate the study variables. Based on reported ethnic background, women were divided into 'white' and 'non-white' groups. Information on ethnicity was not available for about 16% of the study sample. Women with unknown ethnicity were included in the 'white group', as has been performed previously[13] because the

For peer review only - http://bmjopen.bmj.com/site/about/guidelines.xhtml

re-distributed proportions matched more accurately with the estimated ethnic profiles in the UK population census. Maternal records relating to problems during the index pregnancy were used to generate binary variables for antepartum haemorrhage, gestational diabetes and hypertensive disorders of pregnancy. Three binary variables were generated from the history of medical co-morbidities; pre-existing haemoglobinopathies, pre-existing diabetes mellitus and any other medical comorbidities (excluding obesity).

Statistical analysis

We conducted an initial examination of the relationships between the key individual components of the hypothesised pathways (maternal haemoglobin, birthweight-for-gestational age, maternal infection, and stillbirth) using unadjusted linear and logistic regression analysis. Test for deviations from linearity using fractional polynomials did not suggest the presence of significant non-linear associations between haemoglobin at first visit and birthweight-for-gestational age or between maternal infection and haemoglobin concentration. We did not find any significant moderate to strong correlations among the other factors included in the conceptual framework. We tested for plausible interactions between haemoglobin concentration and mother's ethnicity, haemoglobin and BMI, and birthweight-for-gestational age and ethnicity by fitting interaction terms into each of the univariable models that tested the crude associations between the individual pathway components followed by likelihood ratio testing (LR-test). No significant interactions were observed.

We conducted path-analysis[14] to examine the pathways of effect of haemoglobin concentration on stillbirth guided by the theoretical conceptual framework (Figure-1). Path analysis was performed by fitting a set of regression equations under the assumption that the model is not affected by unmeasured confounding[14]. Weighted least squares adjusted for mean and variance was used to estimate the parameters for the model[15]. This estimator with pair-wise deletion is considered to be an efficient and unbiased estimator for models with missing data[16]. Missing information was <2% for most variables, except for BMI and smoking. Three Goodness-of-fit indices, Comparative Fit Index (CFI), χ^2 test for model fit and Root Mean Square Error of Approximation (RMSEA), each related to a specific aspect of the model were used to quantify the degree of correspondence between the model and the data[17 18]. Indirect effects were computed by multiplying the relevant path coefficients. Statistical significance was considered at the 5% level and the analysis was performed using Mplus version 7.

Sensitivity analysis

For peer review only - http://bmjopen.bmj.com/site/about/guidelines.xhtml

Data on index of multiple deprivation (IMD) quintiles, a measure of socioeconomic status, were available only in the Wolverhampton dataset, hence path-analysis was repeated using these data to measure the effect of IMD quintiles on the hypothesised pathways by testing two models, one with IMD quintiles in addition to the other 11 variables and one without. The results did not vary with the inclusion and exclusion of the variable.

RESULTS

In total 76 babies were stillborn in the study population. Details of the characteristics of the study population and their comparison with that of the general population of pregnant women in England are described in a previous paper[5]. Briefly, the median age of pregnant women was 30 years (range 14 to 53 years) and median BMI was 25 kg/m^2 (range 10 to 74 kg/m^2). Nearly half of the women were multiparous (48%), 13% smoked during pregnancy, and 30% belonged to ethnic minority groups. A quarter of the women had one or more pre-existing medical problems, 0·4% had antepartum haemorrhage, 5% were diagnosed with gestational diabetes, 5% had hypertensive disorders of pregnancy, and about 7% had other problems during the index pregnancy.

As shown in Figure-2, there was a statistically significant crude positive linear association between maternal haemoglobin and birthweight-for-gestational age (Coefficient of association β = 0.09; 95% CI 0.04-0.13; p<0.001) and the crude odds of stillbirth decreased by 3% per centile increase in birthweight-for-gestational age (OR=0.97; 95% CI 0.96 to 0.98; p<0.001) (Figure-3). With regard to the components of the second pathway, the crude odds of maternal infection during index pregnancy decreased linearly per unit increase in haemoglobin concentration (OR=0.99; 95% CI 0.98 to 1.00; p=0.026), but the crude odds of stillbirth did not vary significantly by the presence of maternal infection (OR= 0.36; 95% CI 0.05 to 2.58; p=0.309).

The results of the path-analysis are shown in Figure-4. The parameter estimates are the probability coefficients (β), and their magnitude and direction demonstrate the inter-relationships between the variables included in the pathway. As hypothesised, the path-model showed a significant indirect negative effect of maternal haemoglobin at first visit on stillbirth via birthweight-for-gestational age, although the coefficient of association is small. After controlling for potential confounders, a one standard deviation increase in haemoglobin concentration resulted in 0.01 standard deviation decrease in stillbirth mediated via birthweight-for-gestational age. The hypothesised pathway of effect through maternal infection was not significant at p<0.05. After accounting for the effects through the two hypothesised pathways, there was still a significant direct negative effect of maternal

For peer review only - http://bmjopen.bmj.com/site/about/guidelines.xhtml

haemoglobin on stillbirth (β5= -0.12; 95%CI -0.23 to -0.02; p=0.020). In total (direct and indirect effects), a one standard deviation increase in haemoglobin concentration resulted in 0.13 standard deviation decrease in stillbirth (p= 0.020). In addition, we observed significant indirect effects of other known risk factors such as parity, BMI, smoking, gestational diabetes and pre-existing diabetes mellitus on stillbirth via their effects on birthweight-for-gestational age. These associations have been shown in other studies and therefore further validates our model. Pregnancy induced hypertensive disorders and ethnicity were significant confounders. The goodness-of-fit indices showed a good fit between the model and the data.

DISCUSSION

The causal pathways for stillbirth are complex often with multiple risk factors interacting to influence the eventual outcome. We postulated that two pathways were most likely to mediate the effect of maternal haemoglobin during the first trimester on stillbirth: birthweight-for-gestational age and maternal infection. Of these, only birthweight-for-gestational age was found to be statistically significantly mediating the effect. Neither pathway completely explained the effect of haemoglobin on stillbirth as there was a significant residual direct effect of haemoglobin in the first trimester on stillbirth. This suggests that there are other unidentified factors involved in the pathway(s).

While a number of studies have demonstrated low maternal haemoglobin or maternal anaemia to be a risk factor for stillbirth, this study went a step further in delineating the pathways through which maternal haemoglobin could affect stillbirth. In addition to testing known pathways, our study showed that several mechanisms are still unknown and need further investigation. However, it is important to acknowledge that path-models are not causal models and therefore the findings of our study are hypothesis-generating rather than confirmed causal pathways. Inability to adjust for socioeconomic status in the main model was a limitation, but sensitivity analysis using the Wolverhampton data did not materially change the results. We did not have information about the causes of stillbirth in the study population and it is possible that the mechanisms through which haemoglobin affects stillbirth vary by cause. For example, pathways of effect for stillbirth due to congenital anomalies could be different from the pathways for stillbirth as a result of fetal growth restriction.

The observed pathway of effect of maternal haemoglobin on stillbirth mediated via low birthweight-for-gestational age could be explained by a number of factors. In addition to the possibility of haemoglobin exerting its influence through a reduced oxygen tension, there could be other plausible mechanisms through its interaction with nitric oxide (NO), carbon

For peer review only - http://bmjopen.bmj.com/site/about/guidelines.xhtml

monoxide (CO) and carbon dioxide (CO_2) affecting placental circulation leading to fetal growth restriction resulting in stillbirth in some cases[19 20]. However, these factors need to be explored further to generate evidence of biologically plausible mechanisms. It is known that anaemia per se increases the risk of infection, but iron supplementation in iron replete women has also been associated with infectious causes of stillbirth[21]. In our study, the pathway to stillbirth via maternal infection showed no significant relationship leading us to conclude that the haemoglobin effect on stillbirth was not mediated through infection. However, our data included only overt infections reported during pregnancy and it is possible that sub-clinical infections could influence the pathway.

After accounting for the two hypothesised pathways, known confounders and risk factors for stillbirth, there was still a residual direct effect of haemoglobin on stillbirth. This suggests that the relationship between maternal haemoglobin at first trimester and stillbirth cannot be explained with what we already know. This presents the prospect of new and novel mechanisms through which haemoglobin may be affecting the risk of stillbirth. The formation of the placenta from the earliest stages of pregnancy is a highly dynamic process. The early conceptus is a largely hypoxic environment and it is possible that its transition to an oxygen rich environment with a fully functional placenta is adversely affected by low haemoglobin, either through deficient oxygen delivery to the relevant tissues or through maladaptation of the NO driven vascular redistribution through vasodilatation in the presence of hypoxia. However, as well as affecting vascular tone NO has two other important functions, 1) influencing cell signalling and cellular interactions; and 2) neural function[22]. The first two could lead to pathologies that are currently classed histologically as villous dysmaturity, often seen with some normally grown stillbirths, rather than the vasculopathy associated with pre-eclampsia and growth restricted fetuses. Further research matching placental histological findings to cellular function may help to reveal molecular and functional abnormalities that are also critical to normal placental development and fetal survival.

An altogether more prosaic explanation is that the mother's concentration of haemoglobin, or more specifically low haemoglobin, could be a marker for another 'abnormality' (example: undiagnosed inflammatory conditions, autoimmune disease, renal disease, nutritional deficiencies, etc). Iron itself plays a pivotal role in several metabolic processes and a deficiency at any time during a pregnancy may confer a disadvantage on the woman and/or the fetus. Low iron stores, leading to iron deficient anaemia, may be an indicator of poor nutrition and deficiencies in other micronutrients, which either alone or in concert may play a role in the increased risk of stillbirth[23 24]. Finally, epigenetic phenomena affecting gene expression in the maternal genome could be exerting a significant influence on placental and fetal development in the first trimester. The imprinting or silencing of some genes through

For peer review only - http://bmjopen.bmj.com/site/about/guidelines.xhtml

epigenetic mechanisms may adversely affect the foundations laid down in the first trimester and lead to an unrecognised higher risk pregnancy. Iron is a major cofactor for many metabolic processes including imprinting through methylation of sequences of DNA. Other epigenetic mechanisms such as templating, (structural changes to cell membranes), or interfering with RNA silencing also have critical roles to play. Alternatively, rather than iron, these epigenetic mechanisms may be affected by haemoglobin itself via its other suggested functions such as a NO donor[25].

While the antecedents of stillbirth are well-known the mechanisms through which they exert their effect on this outcome still remain unclear. Our findings clearly show that while some of the influence on risk of stillbirth acts through low birthweight-for-gestational age possibly as an adjunct to vascular pathology in the placenta, the majority does not. Haemoglobin may be exerting its influence on the risk of stillbirth involving genetic, epigenetic and/or alternative obstetric and nutritional pathologies, but more research needs to be undertaken to understand these. Our findings suggest that prevention of anaemia will also have a beneficial impact on birthweight which in turn could influence favourably the intergenerational risk of stillbirth. However, more research needs to be performed on causal mechanisms if we are to understand in-depth the pathologies through which maternal haemoglobin affects pregnancies and fetal outcomes.

REFERENCES

1. Flenady V, Koopmans L, Middleton P, et al. Major risk factors for stillbirth in high-income countries: a systematic review and meta-analysis. Lancet 2011;**377**(9774):1331-40.
2. Ray JG, O'Brien TE, Chan WS. Preconception care and the risk of congenital anomalies in the offspring of women with diabetes mellitus. Q J Med 2001;**94**:435-44.
3. Dicker D, Feldberg D, Samuel N, et al. Spontaneous abortions in patients with insulin dependent diabetes mellitus: the effect of pre-conceptual diabetic control. Am J Obstetric Gynecol 1988;**158**:1161-4.
4. Flenady V, Middleton P, Smith GC, et al. Stillbirths: the way forward in high-income countries. Lancet 2011;**377**(9778):1703-17.
5. Nair M, Churchill D, Robinson S, et al. Association between maternal haemoglobin and stillbirth: a cohort study among a multi-ethnic population in England. British Journal of Haematology 2017;**[In Press]**.
6. Greenland S, Pearl J, Robins JM. Causal diagrams for epidemiologic research. Epidemiology 1999;**10**:37-48.
7. Glymour MM. Using causal diagrams to understand common problems in social epidemiology. In: Oakes JM, Kaufman JS, eds. Methods in social epidemiology. San Francisco, USA: Jossey-Bass, 2006.
8. Lawn JE, Blencowe H, Waiswa P, et al. Stillbirths: rates, risk factors, and acceleration towards 2030. The Lancet 2016.
9. Nair M, Choudhury MK, Choudhury SS, et al. The association between maternal anaemia and pregnancy outcomes: a cohort study in Assam, India. BMJ Global Health 2016;**1**:e000026.
10. Haider BA, Olofin I, Wang M, et al. Anaemia, prenatal iron use, and risk of adverse pregnancy outcomes: systematic review and meta-analysis. BMJ 2013;**346**.

For peer review only - http://bmjopen.bmj.com/site/about/guidelines.xhtml

11. Di Mario S, Say L, Lincetto O. Risk factors for stillbirth in developing countries: a systematic review of the literature. Sexually transmitted diseases 2007;**34**(7):S11-S21.
12. Flenady V, Koopmans L, Middleton P, et al. Major risk factors for stillbirth in high-income countries: a systematic review and meta-analysis. The Lancet 2011;**377**(9774):1331-40.
13. Knight M, Kurinczuk JJ, Spark P, et al. Inequalities in maternal health: national cohort study of ethnic variation in severe maternal morbidities. BMJ: British Medical Journal 2009;**338**:b542.
14. Loehlin JC. *Latent variable models: An introduction to factor, path, and structural analysis*: Lawrence Erlbaum Associates Publishers, 1998.
15. Beauducel A, Herzberg PY. On the performance of maximum likelihood versus means and variance adjusted weighted least squares estimation in CFA. Structural Equation Modeling 2006;**13**(2):186-203.
16. Asparouhov T, Muthén B. Weighted least squares estimation with missing data: MplusTechnical Appendix, 2010.
17. Hooper D, Coughlan J, Mullen M. Structural equation modelling: Guidelines for determining model fit. Articles 2008:2.
18. Kenny DA, McCoach DB. Effect of the number of variables on measures of fit in structural equation modeling. Structural equation modeling 2003;**10**(3):333-51.
19. Schechter AN. Hemoglobin research and the origins of molecular medicine. Blood 2017;**12**(10):3927-38.
20. Gladwin MT, Schechter AN, Kim-Shapiro DB, et al. The emerging biology of the nitrite anion. Nature chemical biology 2005;**1**(6):308-14.
21. Brabin L, Brabin BJ, Gies S. Influence of iron status on risk of maternal or neonatal infection and on neonatal mortality with an emphasis on developing countries. Nutrition reviews 2013;**71**(8):528-40.
22. Ignarrow LJ. *Biology and Pathobiology*. San Diego, CA: Academic Press, 2000.
23. Cross JC, Mickelson L. Nutritional influences on implantation and placental development. Nutrition reviews 2006;**64**(5 Pt 2):S12-8; discussion S72-91.
24. Ramakrishnan U, Grant F, Goldenberg T, et al. Effect of women's nutrition before and during early pregnancy on maternal and infant outcomes: a systematic review. Paediatric and perinatal epidemiology 2012;**26 Suppl 1**:285-301.
25. Jablonka E, Lamb MJ. Interacting Dimensions - Genes and Epigenetic Systems (Chapter 7). Evolution in Four Dimensions Genetic, Epigenetic, Behavioural and Symbolic Variation in the History of Life. London, England: The MIT Press, 2014 241-78.

For peer review only

ACKNOWLEDGEMENTS

We would like to acknowledge the contribution of the following people from the Royal Wolverhampton NHS Trust: Alain Rolli, Clinical Scientist, for extracting the haematological data; Laura Gardiner, Clinical Trials Coordinator, Katherine Cheshire, Research Midwife and Julia Icke, Research Midwife, for validating the clinical and haematological data; Bernie Williams IT midwife for extracting the obstetric clinical data. We also thank Marcelo Canda, Business Information Analyst, Women's Services, Guy's and St. Thomas' NHS Foundation Trust for helping with extracting and merging the clinical and haematological data.

Disclosure of interest: The authors declare that they have no competing interests.

Contribution to authorship: MN designed of the study, carried out the data analysis, interpreted the data, and wrote the first draft of the manuscript. DC designed the study, facilitated the process of data extraction from the hospital records, contributed to the data analysis plan and interpretation of the results, and edited the manuscript. SR facilitated the

For peer review only - http://bmjopen.bmj.com/site/about/guidelines.xhtml

process of data extraction from the hospital records, contributed to interpretation of the results and edited the manuscript. CNP contributed to interpretation of the results and edited the manuscript. SS designed the study, contributed to the data analysis plan and data interpretation, and edited the manuscript. MK designed the study, contributed to the data analysis plan, data interpretation, and edited the manuscript.

Details of ethics approval: Ethics approval was not required since this was a secondary analysis of anonymous hospital data.

Funding: Marian Knight is funded by a National Institute for Health Research (NIHR) Research Professorship (NIHR-RP-011-032). The views expressed in this publication are those of the author(s) and not necessarily those of the NHS, the NIHR or the Department of Health. The funding sources had no role in the study, and the researchers were independent from the funders. The funders had no role in study design, data collection and analysis, decision to publish, or preparation of the manuscript.

Data sharing statement: There are no unpublished data from this study. To access the data, please contact the authors - Susan Robinson and David Churchill.

For peer review only

Figure legends

Figure-1: Theoretical conceptual framework of hypothesised pathways of effect of maternal haemoglobin on stillbirth

Figure 2: Association between birthweight-for-gestational age centiles and haemoglobin at first visit

Figure 3: Association between birthweight-for-gestational age centiles and stillbirth

Figure-4: Path model showing the association between maternal haemoglobin and stillbirth

For peer review only - http://bmjopen.bmj.com/site/about/guidelines.xhtml

Figure-1: Theoretical conceptual framework of hypothesised pathways of effect of maternal haemoglobin on stillbirth

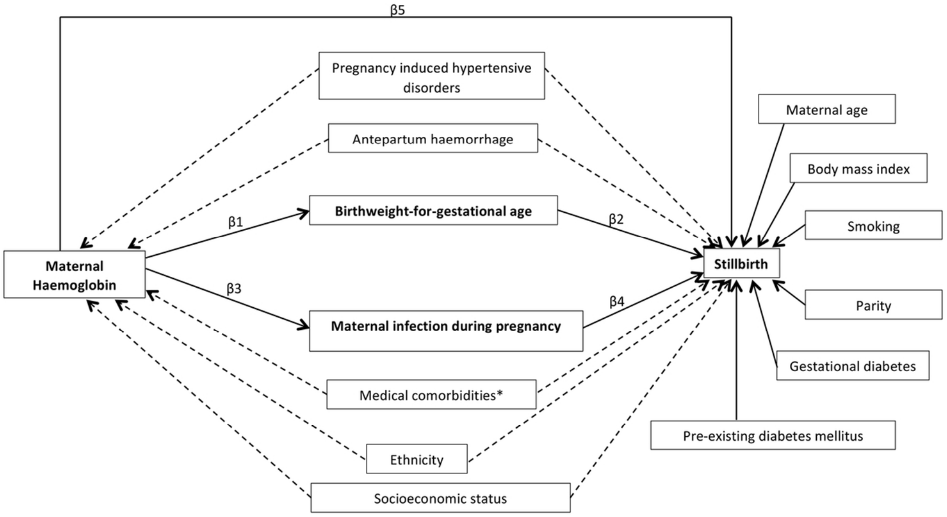

*Medical comorbidities other than pre-existing diabetes mellitus.

The tested pathways are highlighted in bold and confounders are shown using dotted arrows

55x36mm (600 x 600 DPI)

view only

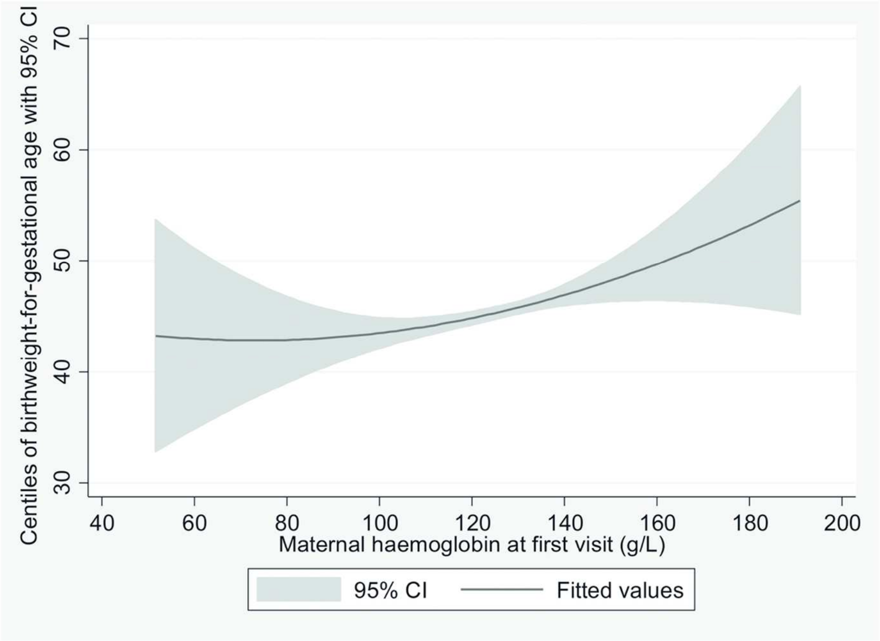

Figure 2: Association between birthweight-for-gestational age centiles and haemoglobin at first visit

64x48mm (600 x 600 DPI)

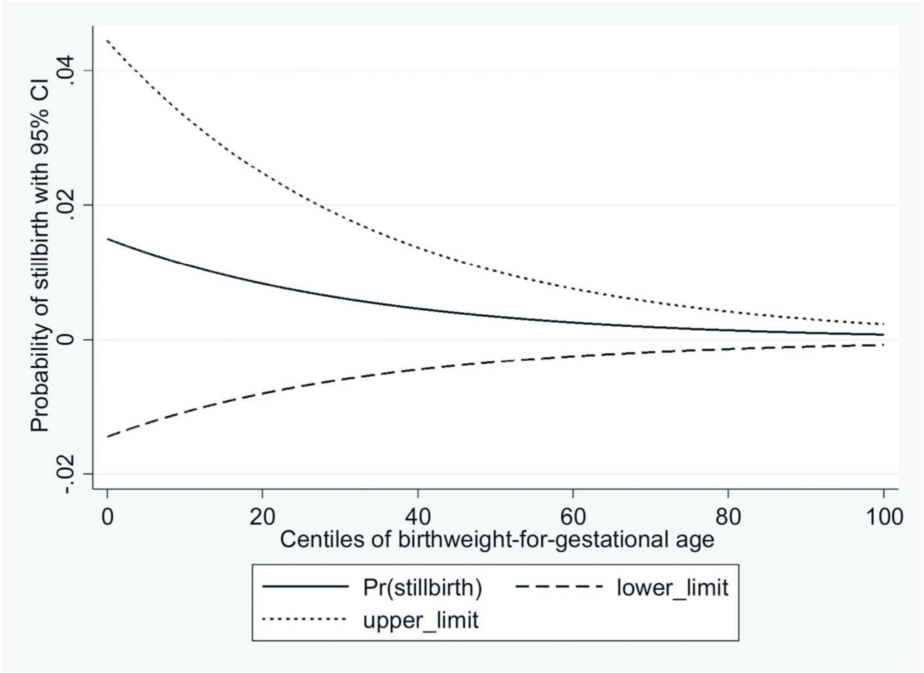

Figure 3: Association between birthweight-for-gestational age centiles and stillbirth

65x50mm (600 x 600 DPI)

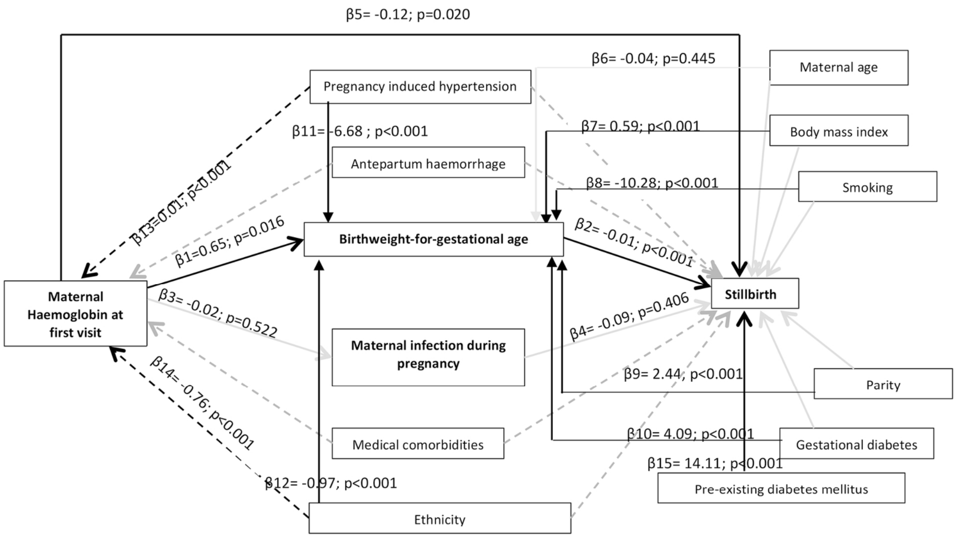

Indirect effect of haemoglobin concentration at first visit on stillbirth via birthweight-for-gestational age: Standardised estimate = -0.01; 95% CI = -0.01 to -0.001; p=0.028. Indirect effect of haemoglobin concentration at first visit on stillbirth via maternal infection: Standardised estimate= 0.001; 95% CI = -0.004 to 0.01; p=0.610. Total indirect effect of haemoglobin concentration at first visit on stillbirth: Standardised estimate= -0.004; 95% CI = -0.01 to 0.003; p=0.267

Total direct and indirect effect of haemoglobin concentration at first visit on stillbirth: Standardised estimate = -0.13; 95% CI = -0.23 to -0.02; p=0.016

P-value for $\chi 2$ test for model fit <0.001; RMSEA = 0.00, 90% CI 0.00 to 0.02; CFI = 1.00; R-Square for stillbirth = 0.09

Figure-4: Path model showing the association between maternal haemoglobin and stillbirth

85x87mm (600 x 600 DPI)

STROBE 2007 (v4) checklist of items to be included in reports of observational studies in epidemiology*
Checklist for cohort, case-control, and cross-sectional studies (combined)

Section/Topic	Item #	Recommendation	Reported on page #
**Title and abstract**	1	(*a*) Indicate the study's design with a commonly used term in the title or the abstract	1 and 2
		(*b*) Provide in the abstract an informative and balanced summary of what was done and what was found	2
**Introduction**			
Background/rationale	2	Explain the scientific background and rationale for the investigation being reported	4
Objectives	3	State specific objectives, including any pre-specified hypotheses	4
**Methods**			
Study design	4	Present key elements of study design early in the paper	5
Setting	5	Describe the setting, locations, and relevant dates, including periods of recruitment, exposure, follow-up, and data collection	5
Participants	6	(*a*) *Cohort study*—Give the eligibility criteria, and the sources and methods of selection of participants. Describe methods of follow-up<br>*Case-control study*—Give the eligibility criteria, and the sources and methods of case ascertainment and control selection. Give the rationale for the choice of cases and controls<br>*Cross-sectional study*—Give the eligibility criteria, and the sources and methods of selection of participants	5 and 6
		(*b*) *Cohort study*—For matched studies, give matching criteria and number of exposed and unexposed<br>*Case-control study*—For matched studies, give matching criteria and the number of controls per case	Not applicable
Variables	7	Clearly define all outcomes, exposures, predictors, potential confounders, and effect modifiers. Give diagnostic criteria, if applicable	5 and 6
Data sources/ measurement	8*	For each variable of interest, give sources of data and details of methods of assessment (measurement). Describe comparability of assessment methods if there is more than one group	5 and 6
Bias	9	Describe any efforts to address potential sources of bias	6 and 7
Study size	10	Explain how the study size was arrived at	5
Quantitative variables	11	Explain how quantitative variables were handled in the analyses. If applicable, describe which groupings were chosen and why	5 and 6
Statistical methods	12	(*a*) Describe all statistical methods, including those used to control for confounding	6 and 7
		(*b*) Describe any methods used to examine subgroups and interactions	6 and 7
		(*c*) Explain how missing data were addressed	6 and 7
		(*d*) *Cohort study*—If applicable, explain how loss to follow-up was addressed<br>*Case-control study*—If applicable, explain how matching of cases and controls was addressed	Not applicable

For peer review only

		Cross-sectional study—If applicable, describe analytical methods taking account of sampling strategy	
		(*e*) Describe any sensitivity analyses	6 and 7
**Results**			
Participants	13*	(a) Report numbers of individuals at each stage of study—eg numbers potentially eligible, examined for eligibility, confirmed eligible, included in the study, completing follow-up, and analysed	7
		(b) Give reasons for non-participation at each stage	Not applicable
		(c) Consider use of a flow diagram	Not applicable
Descriptive data	14*	(a) Give characteristics of study participants (eg demographic, clinical, social) and information on exposures and potential confounders	7
		(b) Indicate number of participants with missing data for each variable of interest	Not applicable
		(c) *Cohort study*—Summarise follow-up time (eg, average and total amount)	Not applicable
Outcome data	15*	*Cohort study*—Report numbers of outcome events or summary measures over time	Not applicable
		Case-control study—Report numbers in each exposure category, or summary measures of exposure	Not applicable
		Cross-sectional study—Report numbers of outcome events or summary measures	Not applicable
Main results	16	(*a*) Give unadjusted estimates and, if applicable, confounder-adjusted estimates and their precision (eg, 95% confidence interval). Make clear which confounders were adjusted for and why they were included	7, 8 and Figures 2,3, 4
		(*b*) Report category boundaries when continuous variables were categorized	Not applicable
		(*c*) If relevant, consider translating estimates of relative risk into absolute risk for a meaningful time period	Not applicable
Other analyses	17	Report other analyses done—eg analyses of subgroups and interactions, and sensitivity analyses	7 and 8
**Discussion**			
Key results	18	Summarise key results with reference to study objectives	8
Limitations	19	Discuss limitations of the study, taking into account sources of potential bias or imprecision. Discuss both direction and magnitude of any potential bias	8
Interpretation	20	Give a cautious overall interpretation of results considering objectives, limitations, multiplicity of analyses, results from similar studies, and other relevant evidence	8, 9 and 10
Generalisability	21	Discuss the generalisability (external validity) of the study results	8, 9 and 10
**Other information**			
Funding	22	Give the source of funding and the role of the funders for the present study and, if applicable, for the original study on which the present article is based	12

*Give information separately for cases and controls in case-control studies and, if applicable, for exposed and unexposed groups in cohort and cross-sectional studies.

Note: An Explanation and Elaboration article discusses each checklist item and gives methodological background and published examples of transparent reporting. The STROBE checklist is best used in conjunction with this article (freely available on the Web sites of PLoS Medicine at http://www.plosmedicine.org/, Annals of Internal Medicine at http://www.annals.org/, and Epidemiology at http://www.epidem.com/). Information on the STROBE Initiative is available at www.strobe-statement.org.

For peer review only

BMJ Open

Pathways of association between maternal haemoglobin and stillbirth: path-analysis of maternity data from two hospitals in England

Journal:	*BMJ Open*
Manuscript ID	bmjopen-2017-020149.R1
Article Type:	Research
Date Submitted by the Author:	20-Feb-2018
Complete List of Authors:	Nair, Manisha; University of Oxford, NPEU, Nuffield Department of Population Health<br>Knight, Marian; National Perinatal Epidemiology Unit<br>Robinson , Susan ; Guy's and St Thomas' NHS Foundation Trust<br>Nelson-Piercy, Catherine; Guy’s & St Thomas’ Foundation Trust<br>Stanworth, Simon; Oxford Radcliffe Hospitals Trust, Department of Haematology/Transfusion Medicine<br>Churchill, David; The Royal Wolverhampton Hospital NHS Trust, New Cross Hospital
**Primary Subject Heading**:	Epidemiology
Secondary Subject Heading:	Public health, Obstetrics and gynaecology
Keywords:	Haemoglobin concentration, pregnancy, stillbirth, path-analysis

SCHOLARONE™
Manuscripts

only

Title page

Pathways of association between maternal haemoglobin and stillbirth: path-analysis of maternity data from two hospitals in England

AUTHORS

1. Manisha Nair
National Perinatal Epidemiology Unit (NPEU), Nuffield Department of Population Health, University of Oxford, Richard Doll Building, Old Road Campus, Headington, Oxford, OX3 7LF, United Kingdom; Email: manisha.nair@npeu.ox.ac.uk

2. Marian Knight
NPEU, Nuffield Department of Population Health, University of Oxford, Richard Doll Building, Old Road Campus, Headington, Oxford, OX3 7LF, United Kingdom, Email: marian.knight@npeu.ox.ac.uk

3. Susan Robinson
Guy's and St Thomas' NHS Foundation Trust
Guy's Hospital, Fourth Floor Southwark Wing, Great Maze Pond, London, SE1 9RT; Email: Susan.Robinson@gstt.nhs.uk

4. Catherine Nelson-Piercy
Guy's and St Thomas' NHS Foundation Trust
10th floor North Wing, St Thomas' Hospital, Westminster Bridge Road, London SE1 7EH; Email: Catherine.Nelson-Piercy@gstt.nhs.uk

5. Simon J Stanworth
Oxford University Hospitals NHS Foundation Trust/ NHS Blood and Transplant
John Radcliffe Hospital, Headley Way, Oxford OX3 9DU; Email: simon.stanworth@nhsbt.nhs.uk

6. David Churchill
The Royal Wolverhampton Hospital NHS Trust, New Cross Hospital, Wolverhampton, WV10 0QP; Email: david.churchill1@nhs.net

Corresponding author

Manisha Nair
National Perinatal Epidemiology Unit (NPEU), Nuffield Department of Population Health, University of Oxford, Richard Doll Building, Old Road Campus, Headington, Oxford, OX3

For peer review only

For peer review only - http://bmjopen.bmj.com/site/about/guidelines.xhtml

7LF, United Kingdom

Phone: 01865-617820

Email: manisha.nair@npeu.ox.ac.uk

ABSTRACT

Objective: To investigate the mechanisms that link maternal haemoglobin concentration with stillbirth.

Design: A retrospective cohort analysis using anonymised maternity data from two hospitals in England.

Setting: The Royal Wolverhampton NHS Trust and Guy's and St Thomas' NHS Foundation Trust

Study population: 12,636 women with singleton pregnancies ≥24 weeks of gestation giving birth in the two hospitals during 2013-15.

Method: A conceptual framework of hypothesised pathways through birthweight-for-gestational age and maternal infection including potential confounders and other risk factors was developed and examined using path-analysis. Path-analysis was performed by fitting a set of regression equations using weighted least squares adjusted for mean and variance. Goodness-of-fit indices were estimated.

Main outcome measures: Coefficient of association (β) for relationship between each parameter, and direct, indirect and total effects via the postulated pathways.

Results: The path-model showed a significant adjusted indirect negative effect of maternal haemoglobin on stillbirth mediated via birthweight-for-gestational age (Standardised estimate (SE) = -0.01; 95% CI = -0.01 to -0.001; p=0.028). The effect through maternal infection was not significant at $p<0.05$ (SE= 0.001; 95% CI = -0.004 to 0.01; p=0.610). There was a residual direct negative effect of maternal haemoglobin on stillbirth (SE = -0.12; 95%CI -0.23 to -0.02; p=0.020) after accounting for the two pathways. Total indirect SE = -0.004; 95% CI -0.01 to 0.003; p=0.267; total direct and indirect SE = -0.13; 95% CI -0.23 to -0.02; p=0.016. The goodness-of-fit indices showed a good fit between the model and the data.

Conclusion: While some of the influence on risk of stillbirth acts through low birthweight-for-gestational age, the majority does not. Several new mechanisms have been suggested for how haemoglobin may be exerting its influence on the risk of stillbirth possibly involving genetic, epigenetic and/or alternative obstetric and nutritional pathologies, but much more research is needed.

ARTICLE SUMMARY

Strengths and limitations of the study

- While a number of studies have demonstrated low maternal haemoglobin to be a risk factor for stillbirth, this study advances the knowledge about the relationship between

For peer review only - http://bmjopen.bmj.com/site/about/guidelines.xhtml

maternal haemoglobin and stillbirth by delineating the pathways using a statistical modelling technique called path-analysis.

- Path-models are not causal models and therefore the findings of this study are hypothesis-generating rather than demonstrating causal pathways.
- Inability to adjust for socioeconomic status in the main model was a limitation, but sensitivity analysis did not materially change the results.
- Information about the causes of stillbirth in the study population was not available and it is possible that the mechanisms through which haemoglobin affects stillbirth vary by cause.

Key words: Haemoglobin concentration, pregnancy, stillbirth, path-analysis

Word count: 2911

For peer review only

For peer review only - http://bmjopen.bmj.com/site/about/guidelines.xhtml

INTRODUCTION

Stillbirth is a global problem, and while the rate has been gradually falling it is difficult to discern the impact of different initiatives. In high income countries, the rates have only marginally improved. Many of the risk factors associated with stillbirth are known and include maternal obesity, advanced maternal age, smoking, small for gestational age fetuses, placental abruptions, placental pathology, pre-existing diabetes and hypertension[1]. Many initiatives have been and are being deployed to try to modify these risk factors and to reduce the rate of stillbirth further, but so far, the improvements have been small, for a combination of reasons. Risk factors such as smoking and obesity are hard to modify in a short period of time and ideally, to have a significant impact, need to be influenced in the peri-conception period. Likewise, in the instance of pre-existing diabetes, it has been shown that pre-conception blood glucose control is as important as good blood glucose control during pregnancy[2 3]. Other risk factors are pathologically non-specific. Small for gestational age, (SGA) refers to a sub-population of fetuses below a set population centile, usually the tenth. Within this group is a sub-set of fetuses that are truly growth restricted, due to placental pathology. These fetuses are at increased risk of adverse obstetric outcome, including stillbirth. In order to identify these 'sick' fetuses, tests of fetal wellbeing need to be performed, for example, Doppler studies of the placental and fetal circulations. While sufficient progress has been made in using ultrasound to identify the genuinely compromised fetus, it is still not 100% sensitive or specific. Risk factors such as fetal growth restriction and abruption are at or near the end point of the natural history of disease and are a manifestation of established placental pathology. As such modifying the processes that lead to fetal death from these stand points is difficult. The main and often only intervention available to the obstetrician is to deliver the baby, which is an intervention that can cause significant morbidity and rarely mortality, for either the mother and/or fetus. As a result, calls have been made to improve the understanding of the epidemiology and the causal pathways of stillbirth so that interventions can be targeted at points where the causes of stillbirth are amenable to modification[4].

We recently studied the association between maternal anaemia and stillbirth in a cohort of 14001 pregnant women[5]. The cohort was drawn from two inner city populations in England. After adjusting for 11 known confounding variables, the risk of stillbirth decreased linearly per unit (10g/l) increase in haemoglobin concentration measured in the first trimester visit between 9 and 12 weeks; aOR = 0.70, 95% CI 0.58-0.85. Compared with women who had a haemoglobin concentration of over 110 g/L in the first trimester, the risk of stillbirth was 5-fold higher in women with moderate to severe anaemia, <100 g/L. The objective of this study

For peer review only - http://bmjopen.bmj.com/site/about/guidelines.xhtml

was to further investigate the mechanisms that link maternal haemoglobin concentration in the first trimester of pregnancy with stillbirth.

METHOD

Study design

We conducted a retrospective cohort analysis using anonymised maternity data from 14,001 women with singleton pregnancies ≥24 weeks of gestation giving birth in two hospitals between 2013 and 2015 (7,175 from Royal Wolverhampton NHS Trust, 2013-14 and 6,826 from Guy's and St Thomas' NHS Foundation Trust, 2014-15). Information on maternal haemoglobin concentration at the first visit, usually between 9 to 12 weeks' gestation was extracted from the hospital laboratory databases and then paired with maternity data. The datasets were then anonymised and analysis was restricted to 12,636 singleton babies born after 24 weeks of gestation and for whom information about haemoglobin at first visit and infant outcomes was available. The outcome 'Stillbirth' was defined as "the death of a baby occurring before or during birth once a pregnancy has reached 24 weeks"[6] A theoretical conceptual framework of the hypothesised pathways through which maternal haemoglobin could be associated with stillbirth was developed using Directed Acylic Graphs[7 8] (shown in Figure 1). This was subsequently tested using a statistical modelling technique known as path-analysis.

Conceptual framework

It has been shown that causal pathways for stillbirth involve fetal growth restriction[9]. Several studies show that maternal haemoglobin concentration is inversely associated with small for gestational age in pregnant women with anaemia[10 11]. Also, low haemoglobin concentration is known to be associated with increased risk of infection during pregnancy and delivery. Therefore, it was hypothesised that the observed effect of haemoglobin concentration on stillbirth could be potentially mediated via birthweight-for-gestational age (an indicator for fetal growth restriction) and/or maternal infection during the index pregnancy. Major factors identified from the literature that could confound the association between maternal haemoglobin and stillbirth were pregnancy induced hypertensive disorders, ethnicity, antepartum haemorrhage, low socioeconomic status, and medical comorbidities. In addition, although not directly associated with maternal haemoglobin, factors such as smoking, high body mass index (BMI), nulliparity, advanced maternal age (>35 years), gestational diabetes and pre-existing diabetes mellitus[9 12 13] are also important as risk factors for stillbirth and therefore were included in the conceptual framework (Figure 1).

For peer review only - http://bmjopen.bmj.com/site/about/guidelines.xhtml

Study variables

Socio-demographic characteristics, BMI, obstetric history, current pregnancy problems, and medical co-morbidities were used to generate the study variables. Based on reported ethnic background, women were divided into ‘white’ and ‘non-white’ groups. Information on ethnicity was not available for about 16% of the study sample. Women with unknown ethnicity were included in the ‘white group’, as has been performed previously[14] because the re-distributed proportions matched more accurately with the estimated ethnic profiles in the UK population census. Maternal records relating to problems during the index pregnancy were used to generate binary variables for antepartum haemorrhage, gestational diabetes and hypertensive disorders of pregnancy. Three binary variables were generated from the history of medical co-morbidities; pre-existing haemoglobinopathies, pre-existing diabetes mellitus and any other medical comorbidities (excluding obesity).

We calculated the z-scores for birthweight-for-gestational age using the LMS-Growth tool that uses Microsoft Excel add-in written using Excel 2000 with Visual Basic for Applications (VBA) based on LMS method and the 1990 British reference cohort. This method adjusts for sex and gestational age while calculating the z-scores. The z-scores were converted to centiles using a standard formula in Excel.

Statistical analysis

We conducted an initial examination of the relationships between the key individual components of the hypothesised pathways (maternal haemoglobin, birthweight-for-gestational age, maternal infection, and stillbirth) using unadjusted linear and logistic regression analysis. Test for deviations from linearity using fractional polynomials did not suggest the presence of significant non-linear associations between haemoglobin at first visit and birthweight-for-gestational age centiles or between maternal infection and haemoglobin concentration. We tested the correlation between the other factors included in the conceptual framework. The calculated pairwise correlation coefficients did not show any statistically significant moderate or strong correlations among the factors. We tested for plausible interactions between haemoglobin concentration and mother’s ethnicity, haemoglobin and BMI, and birthweight-for-gestational age and ethnicity by fitting interaction terms into each of the univariable models that tested the crude associations between the individual pathway components followed by likelihood ratio testing (LR-test). No significant interactions were observed.

We conducted path-analysis[15] to examine the pathways of effect of haemoglobin concentration on stillbirth guided by the theoretical conceptual framework (Figure-1). Path

For peer review only - http://bmjopen.bmj.com/site/about/guidelines.xhtml

analysis was performed by fitting a set of regression equations under the assumption that the model is not affected by unmeasured confounding[15]. Weighted least squares adjusted for mean and variance was used to estimate the parameters for the model[16]. This estimator with pair-wise deletion is considered to be an efficient and unbiased estimator for models with missing data[17]. Missing information was <2% for most variables, except for BMI and smoking. Three Goodness-of-fit indices, Comparative Fit Index (CFI), χ^2 test for model fit and Root Mean Square Error of Approximation (RMSEA), each related to a specific aspect of the model were used to quantify the degree of correspondence between the model and the data[18 19]. Indirect effects were computed by multiplying the relevant path coefficients. Statistical significance was considered at the 5% level and the analysis was performed using Mplus version 7.

Sensitivity analysis

Data on index of multiple deprivation (IMD) quintiles, a measure of socioeconomic status, were available only in the Wolverhampton dataset, hence path-analysis was repeated using these data to measure the effect of IMD quintiles on the hypothesised pathways by testing two models, one with IMD quintiles in addition to the other 11 variables and one without. The results did not vary with the inclusion and exclusion of the variable.

Patient and Public Involvement

This is not applicable since the study was a secondary analysis of anonymous hospital data.

RESULTS

In total 76 babies were stillborn in the study population. Details of the characteristics of the study population and their comparison with that of the general population of pregnant women in England are described in a previous paper[5]. Briefly, the median age of pregnant women was 30 years (range 14 to 53 years) and median BMI was 25 kg/m^2 (range 10 to 74 kg/m^2). Nearly half of the women were multiparous (48%), 13% smoked during pregnancy, and 30% belonged to ethnic minority groups. A quarter of the women had one or more pre-existing medical problems, 0·4% had antepartum haemorrhage, 5% were diagnosed with gestational diabetes, 5% had hypertensive disorders of pregnancy, and about 7% had other problems during the index pregnancy.

As shown in Figure-2, there was a statistically significant crude positive linear association between maternal haemoglobin and centiles of birthweight-for-gestational age (Coefficient of association $\beta = 0.09$; 95% CI 0.04-0.13; $p<0.001$) and the crude odds of stillbirth decreased by 3% per centile increase in birthweight-for-gestational age (OR=0.97; 95% CI 0.96 to 0.98;

For peer review only - http://bmjopen.bmj.com/site/about/guidelines.xhtml

p<0.001) (Figure-3). With regard to the components of the second pathway (association between maternal haemoglobin and stillbirth mediated through maternal infection), the crude odds of maternal infection during current pregnancy decreased linearly per unit increase in haemoglobin concentration (OR=0.99; 95% CI 0.98 to 1.00; p=0.026), but the crude odds of stillbirth did not vary significantly by the presence of maternal infection (OR= 0.36; 95% CI 0.05 to 2.58; p=0.309).

The results of the path-analysis are shown in Figure-4 and coefficients for the direct and indirect pathways are summarised in Table-1. The parameter estimates are the probability coefficients (β), and their magnitude and direction demonstrate the inter-relationships between the variables included in the pathway. As hypothesised, the path-model showed a significant indirect negative effect of maternal haemoglobin at first visit on stillbirth via birthweight-for-gestational age, although the coefficient of association is small. After controlling for potential confounders, a one standard deviation increase in haemoglobin concentration resulted in 0.01 standard deviation decrease in stillbirth mediated via birthweight-for-gestational age. The hypothesised pathway of effect through maternal infection was not significant at p<0.05. After accounting for the effects through the two hypothesised pathways, there was still a significant direct negative effect of maternal haemoglobin on stillbirth (β5= -0.12; 95%CI -0.23 to -0.02; p=0.020). In total (direct and indirect effects), a one standard deviation increase in haemoglobin concentration resulted in 0.13 standard deviation decrease in stillbirth (p= 0.020). In addition, we observed significant indirect effects of other known risk factors such as parity, BMI, smoking, gestational diabetes and pre-existing diabetes mellitus on stillbirth via their effects on birthweight-for-gestational age. These associations have been shown in other studies and therefore further validates our model. Pregnancy induced hypertensive disorders and ethnicity were significant confounders. The goodness-of-fit indices showed a good fit between the model and the data.

Table-1: Direct and indirect pathways of association between maternal haemoglobin at first trimester and stillbirth

Pathways	Coefficient (Standard Error)	P-value
Direct	-0.125 (0.054)	0.020
Total indirect	-0.004 (0.004)	0.267
Total direct and indirect	-0.129 (0.054)	0.016
Specific indirect pathways		
Via birthweight-for-gestational age	-0.005 (0.002)	0.028

For peer review only - http://bmjopen.bmj.com/site/about/guidelines.xhtml

Via maternal infection	0.001 (0.003)	0.610

DISCUSSION

The causal pathways for stillbirth are complex often with multiple risk factors interacting to influence the eventual outcome. We postulated that two pathways were most likely to mediate the effect of maternal haemoglobin during the first trimester on stillbirth: birthweight-for-gestational age and maternal infection. Of these, only birthweight-for-gestational age was found to be statistically significantly mediating the effect. Neither pathway completely explained the effect of haemoglobin on stillbirth as there was a significant residual direct effect of haemoglobin in the first trimester on stillbirth. This suggests that there are other unidentified factors involved in the pathway(s).

While a number of studies have demonstrated low maternal haemoglobin or maternal anaemia to be a risk factor for stillbirth[5 10 20-22], this study went a step further in delineating the pathways through which maternal haemoglobin could affect stillbirth. In addition to testing known pathways, our study showed that several mechanisms are still unknown and need further investigation. However, it is important to acknowledge that path-models are not causal models and therefore the findings of our study are hypothesis-generating rather than confirmed causal pathways. Inability to adjust for socioeconomic status in the main model was a limitation, but sensitivity analysis using the Wolverhampton data did not materially change the results. We did not have information about the causes of stillbirth in the study population and it is possible that the mechanisms through which haemoglobin affects stillbirth vary by cause. For example, pathways of effect for stillbirth due to congenital anomalies could be different from the pathways for stillbirth as a result of fetal growth restriction.

The observed pathway of effect of maternal haemoglobin on stillbirth mediated via low birthweight-for-gestational age could be explained by a number of factors. In addition to the possibility of haemoglobin exerting its influence through a reduced oxygen tension, there could be other plausible mechanisms through its interaction with nitric oxide (NO), carbon monoxide (CO) and carbon dioxide (CO_2) affecting placental circulation leading to fetal growth restriction resulting in stillbirth in some cases[23 24]. However, these factors need to be explored further to generate evidence of biologically plausible mechanisms. It is known that anaemia per se increases the risk of infection, but iron supplementation in iron replete women has also been associated with infectious causes of stillbirth[25]. In our study, the pathway to stillbirth via maternal infection showed no significant relationship leading us to

For peer review only - http://bmjopen.bmj.com/site/about/guidelines.xhtml

conclude that the haemoglobin effect on stillbirth was not mediated through infection. However, our data included only overt infections reported during pregnancy and it is possible that sub-clinical infections could influence the pathway.

After accounting for the two hypothesised pathways, known confounders and risk factors for stillbirth, there was still a residual direct effect of haemoglobin on stillbirth. This suggests that the relationship between maternal haemoglobin at first trimester and stillbirth cannot be explained with what we already know. This presents the prospect of new and novel mechanisms through which haemoglobin may be affecting the risk of stillbirth. The formation of the placenta from the earliest stages of pregnancy is a highly dynamic process. The early conceptus is a largely hypoxic environment and it is possible that its transition to an oxygen rich environment with a fully functional placenta is adversely affected by low haemoglobin, either through deficient oxygen delivery to the relevant tissues or through maladaptation of the NO driven vascular redistribution through vasodilatation in the presence of hypoxia. However, as well as affecting vascular tone NO has two other important functions, 1) influencing cell signalling and cellular interactions; and 2) neural function[26]. The first of the two could lead to pathologies that are currently classed histologically as villous dysmaturity, sometimes seen with some normally grown stillbirths, rather than the vasculopathy associated with pre-eclampsia and growth restricted fetuses. Further research matching placental histological findings to cellular function may help to reveal molecular and functional abnormalities that are also critical to normal placental development and fetal survival.

An altogether more prosaic explanation is that the mother's concentration of haemoglobin, or more specifically low haemoglobin, could be a marker for another 'abnormality' (for example: undiagnosed inflammatory conditions, autoimmune disease, renal disease, nutritional deficiencies, etc). Iron itself plays a pivotal role in several metabolic processes and a deficiency at any time during a pregnancy may confer a disadvantage on the woman and/or the fetus. Low iron stores, leading to iron deficient anaemia, may be an indicator of poor nutrition and deficiencies in other micronutrients, which either alone or in concert may play a role in the increased risk of stillbirth[27 28]. Finally, epigenetic phenomena affecting gene expression in the maternal genome could be exerting a significant influence on placental and fetal development in the first trimester. The imprinting or silencing of some genes through epigenetic mechanisms may adversely affect the foundations laid down in the first trimester and lead to an as yet unrecognised higher risk pregnancy. Iron is a major cofactor for many metabolic processes including imprinting through methylation of sequences of DNA. Other epigenetic mechanisms such as templating, (structural changes to cell membranes), or interfering with RNA silencing also have critical roles to play. Alternatively, rather than iron,

For peer review only - http://bmjopen.bmj.com/site/about/guidelines.xhtml

these epigenetic mechanisms may be affected by haemoglobin itself via its other suggested functions such as a NO donor[29].

While the antecedents of stillbirth are well-known the mechanisms through which they exert their effect on this outcome still remain unclear. Our findings clearly show that while some of the influence from haemoglobin concentration on risk of stillbirth acts through low birthweight-for-gestational age possibly as an adjunct to vascular pathology in the placenta, the majority does not. Haemoglobin may be exerting its influence on the risk of stillbirth involving genetic, epigenetic and/or alternative obstetric and nutritional pathologies, but more research needs to be undertaken to understand these relationships. Our findings suggest that prevention of anaemia will also have a beneficial impact on birthweight which in turn could influence favourably the intergenerational risk of stillbirth. However, more research needs to be performed on causal mechanisms if we are to understand in-depth the pathologies through which maternal haemoglobin affects pregnancies and fetal outcomes.

REFERENCES

1. Flenady V, Koopmans L, Middleton P, et al. Major risk factors for stillbirth in high-income countries: a systematic review and meta-analysis. Lancet 2011;**377**(9774):1331-40.
2. Ray JG, O'Brien TE, Chan WS. Preconception care and the risk of congenital anomalies in the offspring of women with diabetes mellitus. Q J Med 2001;**94**:435-44.
3. Dicker D, Feldberg D, Samuel N, et al. Spontaneous abortions in patients with insulin dependent diabetes mellitus: the effect of pre-conceptual diabetic control. Am J Obstetric Gynecol 1988;**158**:1161-4.
4. Flenady V, Middleton P, Smith GC, et al. Stillbirths: the way forward in high-income countries. Lancet 2011;**377**(9778):1703-17.
5. Nair M, Churchill D, Robinson S, et al. Association between maternal haemoglobin and stillbirth: a cohort study among a multi-ethnic population in England. Br J Haematol 2017;**179**(5):829-37.
6. Manktelow BN, Smith LK, Prunet C, et al. MBRRACE-UK Perinatal Mortality Surveillance Report, UK Perinatal Deaths for Births from January to December 2015. Leicester: The Infant Mortality and Morbidity Studies, Department of Health Sciences, University of Leicester, 2017.
7. Greenland S, Pearl J, Robins JM. Causal diagrams for epidemiologic research. Epidemiology 1999;**10**:37-48.
8. Glymour MM. Using causal diagrams to understand common problems in social epidemiology. In: Oakes JM, Kaufman JS, eds. Methods in social epidemiology. San Francisco, USA: Jossey-Bass, 2006.
9. Lawn JE, Blencowe H, Waiswa P, et al. Stillbirths: rates, risk factors, and acceleration towards 2030. The Lancet 2016.
10. Nair M, Choudhury MK, Choudhury SS, et al. The association between maternal anaemia and pregnancy outcomes: a cohort study in Assam, India. BMJ Global Health 2016;**1**:e000026.
11. Haider BA, Olofin I, Wang M, et al. Anaemia, prenatal iron use, and risk of adverse pregnancy outcomes: systematic review and meta-analysis. BMJ 2013;**346**.
12. Di Mario S, Say L, Lincetto O. Risk factors for stillbirth in developing countries: a systematic review of the literature. Sexually transmitted diseases 2007;**34**(7):S11-S21.
13. Flenady V, Koopmans L, Middleton P, et al. Major risk factors for stillbirth in high-income countries: a systematic review and meta-analysis. The Lancet 2011;**377**(9774):1331-40.

For peer review only - http://bmjopen.bmj.com/site/about/guidelines.xhtml

14. Knight M, Kurinczuk JJ, Spark P, et al. Inequalities in maternal health: national cohort study of ethnic variation in severe maternal morbidities. BMJ: British Medical Journal 2009;**338**:b542.
15. Loehlin JC. *Latent variable models: An introduction to factor, path, and structural analysis*: Lawrence Erlbaum Associates Publishers, 1998.
16. Beauducel A, Herzberg PY. On the performance of maximum likelihood versus means and variance adjusted weighted least squares estimation in CFA. Structural Equation Modeling 2006;**13**(2):186-203.
17. Asparouhov T, Muthén B. Weighted least squares estimation with missing data: MplusTechnical Appendix, 2010.
18. Hooper D, Coughlan J, Mullen M. Structural equation modelling: Guidelines for determining model fit. Articles 2008:2.
19. Kenny DA, McCoach DB. Effect of the number of variables on measures of fit in structural equation modeling. Structural equation modeling 2003;**10**(3):333-51.
20. Rukuni R, Bhattacharya S, Murphy MF, et al. Maternal and neonatal outcomes of antenatal anemia in a Scottish population: a retrospective cohort study. Acta Obstet Gynecol Scand 2016;**95**(5):555-64.
21. Lawn JE, Blencowe H, Pattinson R, et al. Stillbirths: Where? When? Why? How to make the data count? The Lancet 2011;**377**(9775):1448-63.
22. Ali AA, Adam I. Anaemia and stillbirth in Kassala Hospital, Eastern Sudan. Journal of tropical pediatrics 2011;**57**(1):62-4.
23. Schechter AN. Hemoglobin research and the origins of molecular medicine. Blood 2017;**12**(10):3927-38.
24. Gladwin MT, Schechter AN, Kim-Shapiro DB, et al. The emerging biology of the nitrite anion. Nature chemical biology 2005;**1**(6):308-14.
25. Brabin L, Brabin BJ, Gies S. Influence of iron status on risk of maternal or neonatal infection and on neonatal mortality with an emphasis on developing countries. Nutrition reviews 2013;**71**(8):528-40.
26. Ignarrow LJ. *Biology and Pathobiology*. San Diego, CA: Academic Press, 2000.
27. Cross JC, Mickelson L. Nutritional influences on implantation and placental development. Nutrition reviews 2006;**64**(5 Pt 2):S12-8; discussion S72-91.
28. Ramakrishnan U, Grant F, Goldenberg T, et al. Effect of women's nutrition before and during early pregnancy on maternal and infant outcomes: a systematic review. Paediatric and perinatal epidemiology 2012;**26 Suppl 1**:285-301.
29. Jablonka E, Lamb MJ. Interacting Dimensions - Genes and Epigenetic Systems (Chapter 7). Evolution in Four Dimensions Genetic, Epigenetic, Behavioural and Symbolic Variation in the History of Life. London, England: The MIT Press, 2014 241-78.

ACKNOWLEDGEMENTS

We would like to acknowledge the contribution of the following people from the Royal Wolverhampton NHS Trust: Alain Rolli, Clinical Scientist, for extracting the haematological data; Laura Gardiner, Clinical Trials Coordinator, Katherine Cheshire, Research Midwife and Julia Icke, Research Midwife, for validating the clinical and haematological data; Bernie Williams IT midwife for extracting the obstetric clinical data. We also thank Marcelo Canda, Business Information Analyst, Women's Services, Guy’s and St. Thomas’ NHS Foundation Trust for helping with extracting and merging the clinical and haematological data.

Disclosure of interest: The authors declare that they have no competing interests.

For peer review only

For peer review only - http://bmjopen.bmj.com/site/about/guidelines.xhtml

Contribution to authorship: MN designed of the study, carried out the data analysis, interpreted the data, and wrote the first draft of the manuscript. DC designed the study, facilitated the process of data extraction from the hospital records, contributed to the data analysis plan and interpretation of the results, and edited the manuscript. SR facilitated the process of data extraction from the hospital records, contributed to interpretation of the results and edited the manuscript. CNP contributed to interpretation of the results and edited the manuscript. SS designed the study, contributed to the data analysis plan and data interpretation, and edited the manuscript. MK designed the study, contributed to the data analysis plan, data interpretation, and edited the manuscript.

Details of ethics approval: Ethics approval was not required since this was a secondary analysis of anonymous hospital data.

Funding: Marian Knight is funded by a National Institute for Health Research (NIHR) Research Professorship (NIHR-RP-011-032). The views expressed in this publication are those of the author(s) and not necessarily those of the NHS, the NIHR or the Department of Health. The funding sources had no role in the study, and the researchers were independent from the funders. The funders had no role in study design, data collection and analysis, decision to publish, or preparation of the manuscript.

Data sharing statement: There are no unpublished data from this study. To access the data, please contact the authors - Susan Robinson and David Churchill.

Figure legends

Figure-1: Theoretical conceptual framework of hypothesised pathways of effect of maternal haemoglobin on stillbirth

Figure 2: Association between birthweight-for-gestational age centiles and haemoglobin at first visit

Figure 3: Association between birthweight-for-gestational age centiles and stillbirth

Figure-4: Path model showing the association between maternal haemoglobin and stillbirth

For peer review only

Figure-1: Theoretical conceptual framework of hypothesised pathways of effect of maternal haemoglobin on stillbirth

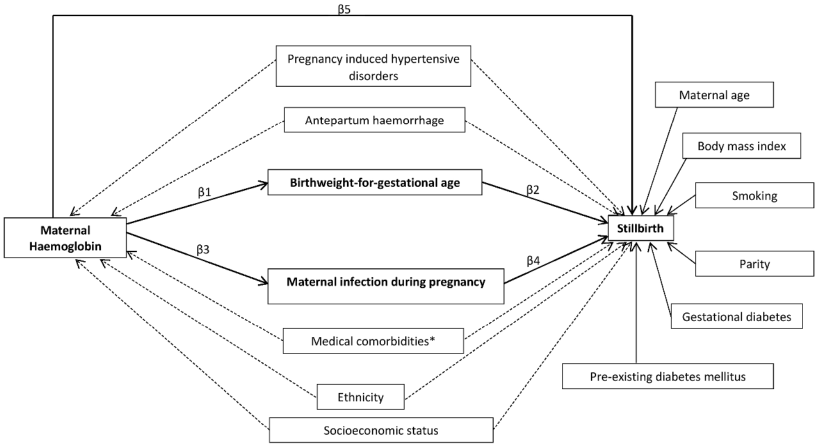

*Medical comorbidities other than pre-existing diabetes mellitus.

The tested pathways are highlighted in bold and confounders are shown using dotted arrows.

B – denotes the 'coefficient of association'

297x209mm (300 x 300 DPI)

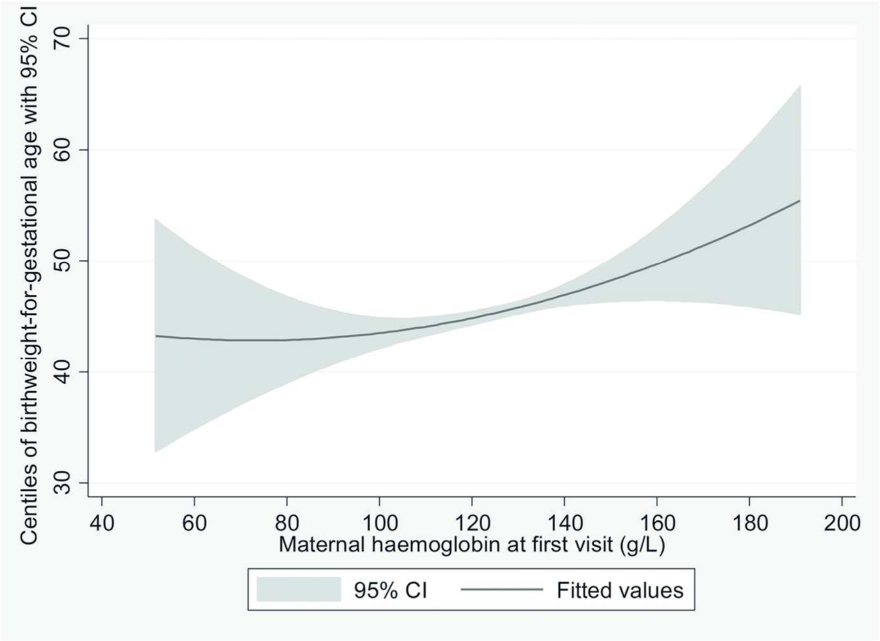

Figure 2: Association between birthweight-for-gestational age centiles and haemoglobin at first visit

64x48mm (600 x 600 DPI)

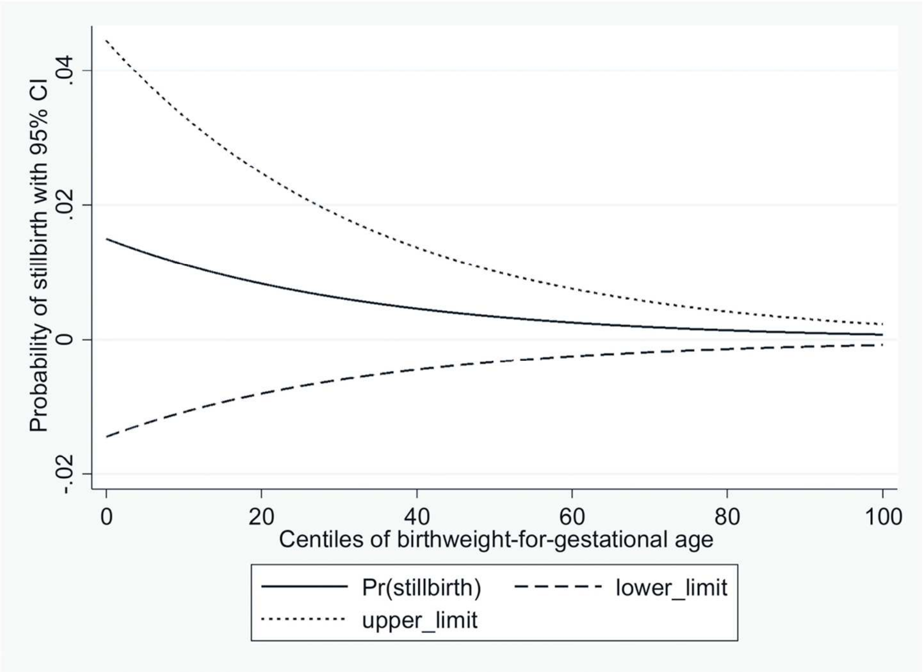

Figure 3: Association between birthweight-for-gestational age centiles and stillbirth

65x50mm (600 x 600 DPI)

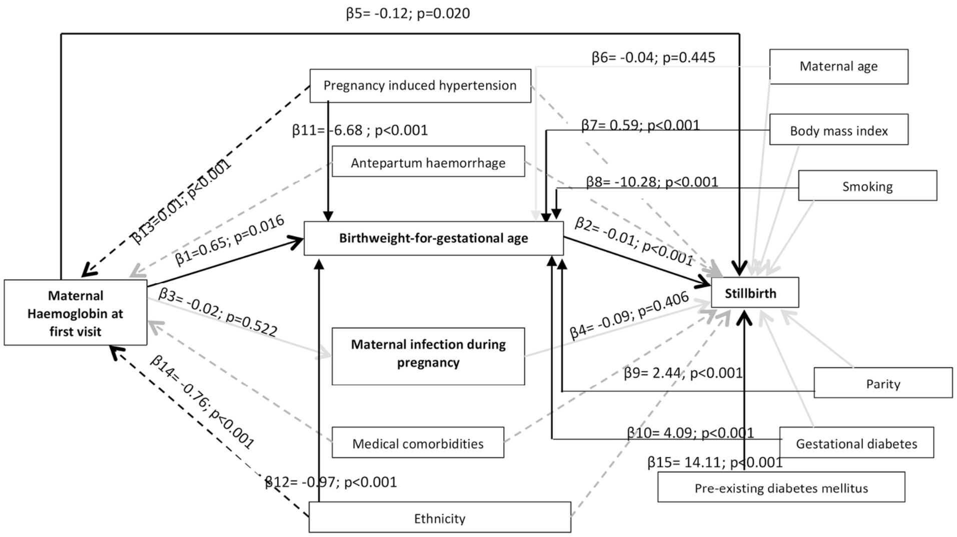

Indirect effect of haemoglobin concentration at first visit on stillbirth via birthweight-for-gestational age: Standardised estimate = -0.01; 95% CI = -0.01 to -0.001; p=0.028. Indirect effect of haemoglobin concentration at first visit on stillbirth via maternal infection: Standardised estimate= 0.001; 95% CI = -0.004 to 0.01; p=0.610. Total indirect effect of haemoglobin concentration at first visit on stillbirth: Standardised estimate= -0.004; 95% CI = -0.01 to 0.003; p=0.267

Total direct and indirect effect of haemoglobin concentration at first visit on stillbirth: Standardised estimate = -0.13; 95% CI = -0.23 to -0.02; p=0.016

P-value for $\chi 2$ test for model fit <0.001; RMSEA = 0.00, 90% CI 0.00 to 0.02; CFI = 1.00; R-Square for stillbirth = 0.09

Figure-4: Path model showing the association between maternal haemoglobin and stillbirth

85x87mm (600 x 600 DPI)

STROBE 2007 (v4) checklist of items to be included in reports of observational studies in epidemiology*

Checklist for cohort, case-control, and cross-sectional studies (combined)

Section/Topic	Item #	Recommendation	Reported on page #
**Title and abstract**	1	(*a*) Indicate the study's design with a commonly used term in the title or the abstract	1 and 2
		(*b*) Provide in the abstract an informative and balanced summary of what was done and what was found	2
**Introduction**			
Background/rationale	2	Explain the scientific background and rationale for the investigation being reported	4
Objectives	3	State specific objectives, including any pre-specified hypotheses	4
**Methods**			
Study design	4	Present key elements of study design early in the paper	5
Setting	5	Describe the setting, locations, and relevant dates, including periods of recruitment, exposure, follow-up, and data collection	5
Participants	6	(*a*) *Cohort study*—Give the eligibility criteria, and the sources and methods of selection of participants. Describe methods of follow-up<br>*Case-control study*—Give the eligibility criteria, and the sources and methods of case ascertainment and control selection. Give the rationale for the choice of cases and controls<br>*Cross-sectional study*—Give the eligibility criteria, and the sources and methods of selection of participants	5 and 6
		(*b*) *Cohort study*—For matched studies, give matching criteria and number of exposed and unexposed<br>*Case-control study*—For matched studies, give matching criteria and the number of controls per case	Not applicable
Variables	7	Clearly define all outcomes, exposures, predictors, potential confounders, and effect modifiers. Give diagnostic criteria, if applicable	5 and 6
Data sources/ measurement	8*	For each variable of interest, give sources of data and details of methods of assessment (measurement). Describe comparability of assessment methods if there is more than one group	5 and 6
Bias	9	Describe any efforts to address potential sources of bias	6 and 7
Study size	10	Explain how the study size was arrived at	5
Quantitative variables	11	Explain how quantitative variables were handled in the analyses. If applicable, describe which groupings were chosen and why	5 and 6
Statistical methods	12	(*a*) Describe all statistical methods, including those used to control for confounding	6 and 7
		(*b*) Describe any methods used to examine subgroups and interactions	6 and 7
		(*c*) Explain how missing data were addressed	6 and 7
		(*d*) *Cohort study*—If applicable, explain how loss to follow-up was addressed<br>*Case-control study*—If applicable, explain how matching of cases and controls was addressed	Not applicable

For peer review only

		Cross-sectional study—If applicable, describe analytical methods taking account of sampling strategy	
		(*e*) Describe any sensitivity analyses	6 and 7
**Results**			
Participants	13*	(a) Report numbers of individuals at each stage of study—eg numbers potentially eligible, examined for eligibility, confirmed eligible, included in the study, completing follow-up, and analysed	7
		(b) Give reasons for non-participation at each stage	Not applicable
		(c) Consider use of a flow diagram	Not applicable
Descriptive data	14*	(a) Give characteristics of study participants (eg demographic, clinical, social) and information on exposures and potential confounders	7
		(b) Indicate number of participants with missing data for each variable of interest	Not applicable
		(c) *Cohort study*—Summarise follow-up time (eg, average and total amount)	Not applicable
Outcome data	15*	*Cohort study*—Report numbers of outcome events or summary measures over time	Not applicable
		Case-control study—Report numbers in each exposure category, or summary measures of exposure	Not applicable
		Cross-sectional study—Report numbers of outcome events or summary measures	Not applicable
Main results	16	(*a*) Give unadjusted estimates and, if applicable, confounder-adjusted estimates and their precision (eg, 95% confidence interval). Make clear which confounders were adjusted for and why they were included	7, 8 and Figures 2,3, 4
		(*b*) Report category boundaries when continuous variables were categorized	Not applicable
		(*c*) If relevant, consider translating estimates of relative risk into absolute risk for a meaningful time period	Not applicable
Other analyses	17	Report other analyses done—eg analyses of subgroups and interactions, and sensitivity analyses	7 and 8
**Discussion**			
Key results	18	Summarise key results with reference to study objectives	8
Limitations	19	Discuss limitations of the study, taking into account sources of potential bias or imprecision. Discuss both direction and magnitude of any potential bias	8
Interpretation	20	Give a cautious overall interpretation of results considering objectives, limitations, multiplicity of analyses, results from similar studies, and other relevant evidence	8, 9 and 10
Generalisability	21	Discuss the generalisability (external validity) of the study results	8, 9 and 10
**Other information**			
Funding	22	Give the source of funding and the role of the funders for the present study and, if applicable, for the original study on which the present article is based	12

*Give information separately for cases and controls in case-control studies and, if applicable, for exposed and unexposed groups in cohort and cross-sectional studies.

Note: An Explanation and Elaboration article discusses each checklist item and gives methodological background and published examples of transparent reporting. The STROBE checklist is best used in conjunction with this article (freely available on the Web sites of PLoS Medicine at http://www.plosmedicine.org/, Annals of Internal Medicine at http://www.annals.org/, and Epidemiology at http://www.epidem.com/). Information on the STROBE Initiative is available at www.strobe-statement.org.

For peer review only
